# Dietary Plasticity of Generalist and Specialist Ungulates in the Namibian Desert: A Stable Isotopes Approach

**DOI:** 10.1371/journal.pone.0072190

**Published:** 2013-08-16

**Authors:** David Lehmann, John Kazgeba Elijah Mfune, Erick Gewers, Johann Cloete, Conrad Brain, Christian Claus Voigt

**Affiliations:** 1 Leibniz Institute for Zoo and Wildlife Research (IZW), Berlin, Germany; 2 University of Namibia (UNAM), Department of Biological Sciences, Windhoek, Namibia; 3 Torra Community-Based Conservancy Office, Kunene Region, Namibia; 4 Wilderness Safaris Namibia, Windhoek, Namibia; Bangor University, United Kingdom

## Abstract

Desert ungulates live in adverse ecosystems that are particularly sensitive to degradation and global climate change. Here, we asked how two ungulate species with contrasting feeding habits, grazing gemsbok (*Oryx g. gazella*) and browsing springbok (*Antidorcas marsupialis*), respond to an increase in food availability during a pronounced rain period. We used a stable isotope approach to delineate the feeding habits of these two ungulates in the arid Kunene Region of Namibia. Our nineteen months field investigation included two time periods of drought when food availability for ungulates was lowest and an intermediate period with extreme, unusual rainfalls. We documented thirteen isotopically distinct food sources in the isotopic space of the study area. Our results indicated a relatively high dietary plasticity of gemsbok, which fed on a mixture of plants, including more than 30% of C3 plants during drought periods, but almost exclusively on C4 and CAM plant types when food was plentiful. During drought periods, the inferred gemsbok diets also consisted of up to 25% of *Euphorbia damarana*; an endemic CAM plant that is rich in toxic secondary plant compounds. In contrast, springbok were generalists, feeding on a higher proportion of C3 than C4/CAM plants, irrespective of environmental conditions. Our results illustrate two dietary strategies in gemsbok and springbok which enable them to survive and coexist in the hostile Kunene arid ecosystem.

## Introduction

Ungulate species have colonized a wide range of habitats worldwide with different and variable food resources. They even manage to inhabit areas of extreme environmental conditions such as dry savannahs and deserts [1], [2]. The survival of ungulates in these specific environments depends on the abundance of adequate food plants that offer sufficient nutrients for survival [3]. The availability of these resources often varies with time, and ungulates therefore may face challenges when undergoing a resource bottleneck. Largely, ungulates may rely on one of two strategies when facing reduced resource availability: either they stay and broaden their diet or they leave in search of their specialized, but temporarily restricted diet [4]. The use of these strategies depends, at least partly, on the intrinsic degree of dietary specialization, which is thought to be inversely related to body size [5], [6], [7], [8]. Following these predictions, small antelopes display pronounced dietary specificity with low tolerance to changes in selected food availability, whereas larger ungulates are more plastic in their feeding habits and thus dietary generalist.

Small-sized antelopes with high metabolic rates and a relatively short digestive tract with low food retention capacity tend to specialize on highly nutritive and digestible food items, since they are not well suited to internally process food sources of poor quality (e.g. dry forage) [7], [8], [9]. A high dietary specificity of small body-sized ungulates has been suggested for a variety of smaller ungulates. For example the African duikers *Cephalophus monticola* and *Cephalophus maxwelli* are specialized exclusively on a diet of fruits and stems which have low fibre content and high digestibility [10]. However, new studies have shown that some small ruminant species are able to significantly broaden their diet when needed. For example, the Cape grysbok (*Raphicerus melanotis*) is usually a highly selective browser feeding mostly on *Acacia* leaves [11]. Nevertheless this species may broaden its feeding behaviour temporarily to include grass and previously rejected plant species when Acacias were removed from the environment [12]. Other small ruminant species such as springbok (*Antidorcas marsupialis*) are mixed feeders and include grass, succulent plants and leaves of shrubs in their diet [13], [14], [15]. Additionally, they may adjust their diet in response to the availability of food sources with variable quality, preferring for example grass sprouts during the wet season [16], [17], and browsing predominantly on leaves of bushes when grass quality decreases [18].

This pattern of variable food selection has also been reported in a wide range of larger ungulates, which should not be constrained in their dietary choice by body size but by their quantitative requirements. Indeed, vicuña (*Vicugna vicugna*), white-tailed deer (*Odocoileus virginianus)* and historic populations of bison (*Bison antiquus*) show dietary plasticity and generalization when their preferential food plants are scarce [19], [20], [21]). Gemsbok (*Oryx gazella gazella*) of the African savannah have been categorized as grazers or hyper-grazers, which implies that despite their large body size they specialize on grasses (up to 85% to 100% of their diet [22], [23]. Nonetheless, studies on gemsbok populations of the Kalahari show that they browse as well on succulent plants and also eat underground tubers (such as the so-called gemsbok cucumber *Acanthosicyos naudinianus*) during the dry season [24]. The ability to optionally use these alternative food sources may be crucial in arid environments although the high content of secondary plant compounds of these plants may hamper the digestive efficiency [8], [25], [26].

Here, we investigated the link between temporal environmental changes and feeding habits in two desert ungulate species. A knowledge about the plasticity of ungulate dietary strategies is especially important since climate change is expected to promote desertification [27], to cause loss in primary productivity and consequently to facilitate species extinction [28]. We therefore asked how antelope species respond to changes in food availability in semi-desert ecosystems.

Here, we quantitatively estimated the plasticity of feeding habits in gemsbok and springbok, which are the most abundant ungulates in the southern part of the arid Kunene region of Namibia. This environment is characterized by strong and unpredictable variation in resource availability [29]. We hypothesised that springbok and gemsbok rely on different food sources and display different patterns of resource use over time. As previous studies suggested that gemsbok are specialist grazers and springbok generalist feeders [1], [2], we predicted that springbok respond more strongly in their dietary niche than gemsbok when facing a shortage of food sources. We predicted that during drought years springbok include food items that have been avoided during periods of good primary productivity. We used stable carbon and nitrogen isotope ratios to provide quantitative information on the relative contribution of different food resources to the diet of gemsbok and springbok across nineteen months, corresponding to one and a half seasonal cycle, of variable resource availability [23], [30]. Many desert plants differ in their stable carbon isotope ratios depending on their metabolic pathway of CO_2_ fixation, which is either the C4 and CAM (grasses and *Euphorbia* respectively) or the C3 (trees and shrubs) photosynthetic pathway [31], [32], [33]. Since stable isotope ratios in tissues of gemsbok and springbok vary according to whether they consume grass, euphorbia, leaves of trees and shrubs or a combination of them, we used a stable isotope approach in our study. In particular, we analysed stable carbon and nitrogen isotope ratios to provide quantitative information on the relative contribution of food resources to the diet of gemsbok and springbok across three years of variable resource availability. The information gathered in this study might shed light on the mechanisms of resource partitioning between these two species and provide important knowledge for local wildlife management plans.

## Methods

### Study Site

This study took place from November 2010 to June 2012 in the southern part of the Kunene region of Namibia (−20° 12′ 58.59′′ N, +14° 4′ 6.24′′ E), a 3,500 km^2^ area that is managed by the Torra Conservancy under the premise of sustainable use of natural resources. Gemsbok and springbok represent one of the main protein sources from wildlife for local communities. The local ecosystem consists of outcrops of basaltic mountain ranges, rocky and gravel plains, dry riverbeds and deltas formed by the ephemeral Huab and Springbok rivers. The annual precipitation usually ranges between 100 and 150 mm and the ambient temperature may reach up to 50°C during the dry season [29]. In 2010, the area received less than 80 mm of rain while in 2011 it received more than 500 mm (Torra conservancy, Damaraland Camp Weather station, [34]). In 2012, annual precipitation returned to less than 180 mm of rainfall (Torra conservancy, Namibian Weather Network [34]). Vegetation ranges from scarce open grasslands with bushes to plain rocky and/or sandy desert [29].

### Study Species

Gemsbok are large ungulates (180 to 240 kg) that have a geographical distribution from South Africa to northern Namibia and the southern parts of Botswana [2]. Springbok are smaller in body size (30 to 44 kg) and occur from the northwestern part of South Africa through the Kalahari Desert into Namibia and Botswana [2]. The distribution range of both gemsbok and springbok includes various habitats including savannahs, woodlands and deserts [2].

### Sample Collection

Our work was carried out under the Research Permits number 1534/2010 and 1676/2012 issued by the Ministry of Environment and Tourism of Namibia and has been approved by the Internal Committee for Ethics and Animal Welfare of the Leibniz Institute for Zoo and Wildlife Re-search (IZW) of Berlin. Also, the Torra Conservancy had granted us the right to conduct our research on the Torra Conservancy communal land, on behalf of the conservancy members.

We collected and analysed the stable isotope ratios of potential food sources and three types of tissues (blood, liver, muscle) from both species. We first collected plant and animal tissues samples from November 2010 to February 2011, which corresponded to a period of severe drought, labelled as “2010” in our study. We continued our sample collection from April 2011 to August 2011, time period associated with heavy rainfalls and increased primary productivity. This time period was labelled “2011” in our study. Our third fieldwork session was performed from February 2012 to June 2012, which corresponds to another drought period, labelled as “2012” in our study.

Collected plant species were selected according to the known diet of the springbok and gemsbok [2], direct observations or based on information from local game guards. We also collected samples from plants from which feeding observations by ungulate were not recorded and from plants of poor availabilities, scarcely distributed in the environment. From each plant, we collected about 2 g wet mass of leaves. Samples were dried in the sun before storing them in 1.5 ml Eppendorf vials. We obtained samples from the following species; Trees (C3): *Colophospermum mopane* (leaves; n = 26), *Boscia albitrunca* (leaves; n = 26), *Faidherbia albida* (leaves) (n = 14), *Acacia erioloba* (leaves, flowers and pods; n = 27), *Acacia tortilis* (leaves, flowers and pods; n = 33), *Terminalia prunoides* (fruits and leaves; n = 22), *Tamarix usneoides* (leaves; n = 18); Bushes and shrubs (C3): *Calicorema capitata* (leaves and flowers; n = 14), *Boscia foetida* (leaves; n = 25), *Salvadora persica* (leaves; n = 13), *Cyperus marginatus* (leaves; n = 34), *Petalidium spiniferum* (leaves; n = 39), *Cadaba shroepelli* (leaves*;* n = 35), *Phaeoptilum spinosum* (leaves; n = 18)*;* Grasses (C4): *Stipagrostis damarensis* (n = 12), other *Stipagrostis sp.* (n = 42) and *Eragrostis sp.* (n = 53) and succulent plants (CAM): *Euphorbia damarana* (fruits and branches; n = 26), *Salsola sp* (leaves; n = 11) and *Zygophylum simplex* (leaves; n = 11). Additionally, we collected material from a few ephemeral bushes and flowers during the unusually heavy rainy season of 2011, including samples of *Petalidium halimoides* (n = 10), *Pegolettia oxydonta* (n = 6), *Geigera alata* (n = 6), *Trichodesma africanum* (n = 6), *Lotononis sp.* (n = 6), *Cleome foliosa* (n = 6) and *Heliotropium oliveranum* (n = 6) as well as *Indigofera adenocarpa* (n = 6). Samples from the same species were collected from various habitats such as riverbeds, plains, valleys, hills and mountain slopes to cover most of the important habitats of the area in which gemsbok and springbok populations lived. In total, we collected 551 plant samples from 29 species.

Tissues of springbok and gemsbok (herein after referred to as consumer tissues or materials) were collected during the communal hunting of the Torra community-based conservancy or from fresh carcasses found in the field. Animals were not killed for the purpose of this study; we were granted the right to collect samples from hunt products by the Torra Conservancy. Under Namibia’s Community Based Natural Resource Management Program [35], conservancies are given annual quotas to kill selected mammals as a source of revenue for community projects and food to households. During the study period, we collected samples (2 g muscle tissue, 2 g liver tissue and 1 ml blood) from 56 gemsbok and 55 springbok individuals. From gemsbok, we collected samples from 6 males and 3 females in 2010, from 18 males and 20 females in 2011 and from 4 males and 5 females in 2012. From springbok, we collected samples from 2 males and 4 females in 2010, from 17 males and 26 females in 2011 and from 4 males and 2 females in 2012. All samples were dried in the sun and then stored in 1.5 ml plastic vials. Plant and tissue samples were shipped to the stable isotope laboratory at the Leibniz Institute for Zoo and Wildlife Research in Berlin, Germany. In the laboratory, all samples were washed with distilled water, dried in an oven (Heraeus Function Lab, Thermo Electron Corporation, 63505 Langensbold, Germany) until constant mass, and then powdered using a mortar grinder (RETSCH GmbH milling machine). An aliquot sample of 1.5 and 2 mg and 0.3–0.4 mg, for plant and consumer materials respectively were loaded into tin capsules (COSTECH Analytical Inc.).

Samples were combusted and the resultant gases (N_2_ and CO_2_) were sequentially measured in a CE 1110 elemental analyzer connected via a continuous flow system to a Thermo Finnigan Delta Plus isotope ratio mass spectrometer (Thermo Finnigan, Bremen, Germany). The sample isotope ratios were compared with international gas standards (USGS-24 and IAEA-N1) [36]. Isotope ratios were expressed in the δ notation in parts per thousand (‰) [37]. We used stable carbon isotope ratio of Vienna Pee Dee Belemnite limestone and the nitrogen isotope ratio of air as reference. The precision of measurements was better than 0.1‰ (one standard deviation) for both elements.

### Statistical Analysis

**Figure 1:**
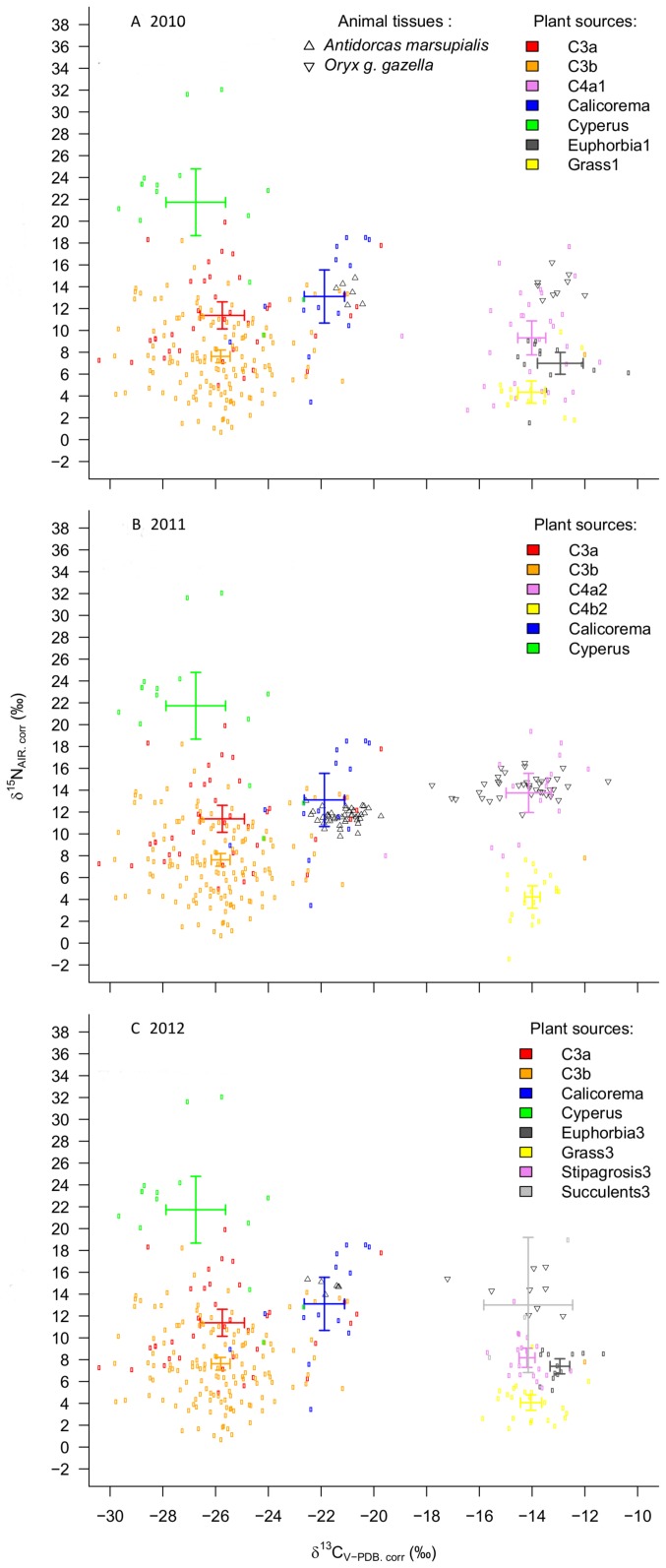
Isotopic projections along the nitrogen and carbon axes (all in delta notation) of the thirteen major potential food sources (Mean; 95%CI) in A) 2010, B) 2011 and C) 2012 plotted with the mean values of the three metabolically active tissues (blood, liver and muscle) of gemsbok and springbok sampled in A) 2010, B) 2011 and C) 2012.

To delineate the feeding habits of gemsbok and springbok, we used isotope mixing models that included information about δ^13^C and δ^15^N for both consumer and food material [38], [39], [40]. We also controlled for variable concentrations of C and N in focal plants [41], [42], [43], [44], [45]. The use of stable isotopic techniques to study animal diets requires a priori estimates of isotope discrimination within the tissues of interest (Δ^ 13^C and Δ^ 15^N, also called discrimination factors), which represent the differences in isotopic composition between animal tissues and the animals’ diet. Since we did not know the species-specific discrimination factors for our study species, we referred to discrimination factors for blood, liver and muscle of mammalian species according to Caut and colleagues [46] for blood and liver, and to the work of Sutoh [47] and Codron and colleagues [38] for muscle. Hence, we corrected raw stable isotopic ratios of carbon and nitrogen of blood by −1.3 ‰ and 2.7 ‰, of liver by −0.69 ‰ and 3.3 ‰, of muscle by 1.5 ‰ and 2.9‰ (Δ^ 13^C and Δ^ 15^N, respectively). We used the Monte Carlo Mapped Power (MCMP) isotope mixing models from the package SIAR version 4.1.3 [48] of the free statistical software R (R Development Core Team, 2010) to assess the relative contribution of plant resources to the diet of our two study species. Furthermore, we report the estimated proportions of food sources to the gemsbok and springbok diets given by the most likely source mixture solutions calculated by the Bayesian isotope mixing models [39], [43], [49]. The range of variation in ‰ was sufficiently high to warrant a high resolution in the estimate of the plants’ relative contribution to the overall diet of gemsbok and springbok, e.g. >10 ‰ in both δ^15^N and δ^13^C (Fig. 1) [50]. We selected *a priori* eleven plant groups as potential food sources and categorized them according to their photosynthetic pathway and their similarity or apparent differences in stable carbon and nitrogen isotope ratios. The potential food sources were clustered as followed: “*Stipagrostis*” (n = 54) and “Grass” (n = 53) for the C4 plant type; “*Euphorbia*” (n = 26) and “Other succulents” (n = 22) for the CAM plant group and “*Calicorema*” (n = 14), “Weed & others” (n = 14), “Shrub” (n = 78), “Tree” (n = 52), “*Boscia*” (n = 16), “*Salvadora*” (n = 13), “*Cyperus*” (n = 27) for the C3 plant group, which summed up to 369 plant samples used.

Since we sampled plant species or plant groups during three field trips over a period of nineteen months in the same environment, we performed a MANOVA, including “year” as fixed factor, for C3, C4 and CAM plant categories separately, to assess whether the isotopic composition of each clustered food resources differed between years and between them. We then performed a pairwise comparison MANOVA, excluding the year of sampling as co-parameter and including plant groups as factor, to test whether these defined groups were isotopically different.

Using MANOVA, with “year” included as fixed factor, we tested whether the isotopic composition of the two species differed and whether it varied among years. We performed Mann Whitney pairwise comparisons to delineate between the potential effects of the different years of sampling on the mean tissues stable carbon and nitrogen isotopic compositions of gemsbok and springbok.

## Results

### Plant Stable Isotope Composition

In general, δ^13^C of plants followed a bimodal distribution, consistent with the isotopic contrast between C3, C4 and CAM plants (Table 1). δ^15^N values were more evenly distributed (Table 1). Stable carbon and nitrogen isotope ratios of potential feeding plants with a C3 photosynthetic pathway did not differ among years (F_2,203_ = 0.37; Pillai = 0.007; p = 0.83) but differed between a priori defined plant categories (F_6,203_ = 19.6; Pillai = 0.73; p<0.001). These differences were consistent over years (F_11,203_ = 2.9; Pillai = 0.27; p<0.001). We therefore pooled data of different years for each element and plant category in this C3 plant group.

**Table 1:** Summary of the Mean ± s.d. values of δ^ 13^C and δ^15^N for thirteen food sources.

Source (n = 369)	Type	“Year”	δ^13^C (Mean ± s.d.) (‰)	δ^15^N (Mean ± s.d.) (‰)
C3a (n = 43)	C3	2010, 11 & 12	−25.6±2.2	11.5±4.8
C3b (n = 130)	C3	2010, 11 & 12	−25.9±1.9	7.8±3.9
*Calicorema* (n = 14)	C3	2010, 11 & 12	−21.4±2.2	13.1±4.5
*Cyperus* (n = 27)	C3	2010, 11 & 12	−26.5±3.3	20.8±6.9
C4a1 (n = 31)	55% C4–45% CAM	2010	−14±1.8	9.3±1.8
Grass1 (n = 16)	C4	2010	−14±1	4.3±1.2
*Euphorbia*1 (n = 15)	CAM	2010	−12.9±1.7	7±1.1
C4a2 (n = 16)	56% C4–44% CAM	2011	−14.1±1.7	13.7±1.3
C4b2 (n = 19)	84% C4–16% CAM	2011	−13.9±0.6	4.2±0.7
Stipagrostis3 (n = 19)	C4	2012	−14.1±0.6	8.2±1.2
Other succulents3 (n = 3)	CAM	2012	−14.1±1.5	13±5.4
Grass3 (n = 23)	C4	2012	−14.0±0.9	4.1±1.1
Euphorbia3 (n = 13)	CAM	2012	−12.9±0.7	7.4±1

We observed that “*Boscia*” and “*Salvadora*” had similar isotopic signatures (F_1,35_ = 0.66; Pillai = 0.037; p = 0.52). “*Boscia*” and “*Salvadora*” also shared a similar isotopic signature with “Weed & others” (F_1,37_ = 0.06; Pillai = 0.0033; p = 0.94 and F_1,26_ = 019; Pillai = 0.015; p = 0.82; respectively). Stable isotope ratios of the categories “Tree” and “Shrub” were similar (F_1,140_ = 2.65; Pillai = 0.037; p = 0.073). “*Boscia*” and “Tree” differed in their isotopic composition (F_1,85_ = 5.33; Pillai = 0.11; p = 0.0066). Moreover, “Tree” differed from “*Salvadora*” (F_1,74_ = 5.37; Pillai = 0.13; p = 0.0066) and “Weed & others” (F_1,101_ = 14.45; Pillai = 0.22; p<0.001). “*Boscia*” differed from “Shrub” (F_1,76_ = 4.56; Pillai = 0.11; p = 0.013). Hence, we grouped “*Boscia*”, “*Salvadora*” and “Weed & others” in the category “C3a” (n = 43) and “Shrub” and “Tree” in the category “C3b” (n = 130). Following this, the isotopic mixture of “C3a” differed from “C3b” (F_1,192_ = 21.11; Pillai = 0.18; p<0.001), from “*Calicorema*” (F_1,64_ = 15.68; Pillai = 0.33; p<0.001) and “*Cyperus*” (F_1,65_ = 33.7; Pillai = 0.33; p<0.001). “C3b” also formed an independent cluster from “*Calicorema*” (F_1,154_ = 32.8; Pillai = 0.3; p<0.001) and “*Cyperus*” (F_1,155_ = 90.47; Pillai = 0.54; p<0.001). Lastly, “*Calicorema*” and “*Cyperus*” were found to be significantly different (F_1,27_ = 12.24; Pillai = 0.48; p<0.001) (Fig. 1).

Our MANOVA analysis revealed that stable carbon and/or nitrogen isotope ratios of C4 potential plant sources differed between years (F_2,143_ = 3.47; Pillai = 0.092; p = 0.009) and between *a priori* defined plant categories (F_3,143_ = 22.49; Pillai = 0.64; p<0.001). Our analysis indicated that these differences were consistent over years (F_6,143_ = 2.43; Pillai = 0.18; p = 0.005). We therefore performed MANOVA pair-wise comparisons to test if these C4 and CAM defined groups were isotopically different within years. Moreover, when comparing C4 and CAM plant categories for 2010, we found that “*Stipagrostis*” had a similar isotopic composition compared with “Other succulents” (F_1,29_ = 0.14; Pillai = 0.010 p = 0.86) (Fig. 1A). Consequently, we pooled these two potential food sources as “C4a1” (n = 31) and recalculated our model (Fig. 1A). According to this analysis, “C4a1” differed from “Grass” (F_1,45_ = 9.50; Pillai = 0.30; p = 0.0004) and “*Euphorbia*” (F_1,44_ = 5.05; Pillai = 0.19; p-value = 0.011). “*Euphorbia*” and “Grass” also differed from each other (F_1,29_ = 7.82; Pillai = 0.35; p = 0.002) (Fig. 1A). For 2011, we found that “*Stipagrostis*” had a similar isotopic composition with “Other succulents” (F_1,14_ = 3.40; Pillai = 0.34 p = 0.065) and that ”*Euphorbia*” and “Grass” also shared similar isotopic compositions. Consequently, we pooled “*Stipagrostis*” and “other succulents” potential food sources as “C4a2” (n = 16) and “*Euphorbia*” and “Grass” as “C4b2” (n = 19). Plants of the category “C4a2” differed from those of “C4b2” (F_1,33_ = 62.85; Pillai = 0.79; p<0.0001) (Fig. 1B). In 2012, “*Stipagrostis*” differed from “Other succulents” (F_1,20_ = 4.56; Pillai = 0.32 p = 0.024), from “Grass” (F_1,40_ = 25.68; Pillai = 0.56; p<0.001) and “*Euphorbia*” (F_1,30_ = 12.75; Pillai = 0.46; p = 0.00010). The isotopic composition of “Grass” differed also from “Other succulents” (F_1,24_ = 21.30; Pillai = 0.64; p<0.0001) and from “*Euphorbia*” (F_1,34_ = 23.13; Pillai = 0.58; p<0.0001). “Other succulents” and “*Euphorbia*” differed from each other as well (F_1,14_ = 27.60; Pillai = 0.80; p<0.0001) (Fig. 1C).

To summarize, categories “C3a”, “C3b”, “*Calicorema*”, “*Cyperus*” and “C4a1”, “Grass1”, “*Euphorbia*1” (“2010”; Fig. 1A). “C4a2” and “C4b2” (“2011”; Fig. 1B) and “*Stipagrostis*3”, “Grass3”, “Other succulents3” and “*Euphorbia*3” (“2012”; Fig. 1C) differed significantly in their mean stable carbon and nitrogen isotopes ratios (Table 1). Thus, we documented thirteen resources with distinct mean stable carbon and/or nitrogen isotopes values (Table 1; Fig. 1). Mean values and standard deviations for stable isotope ratios were used in our stable isotope mixing models (Table 1).

### Isotopic Composition of Consumer Tissue

**Figure 2:**
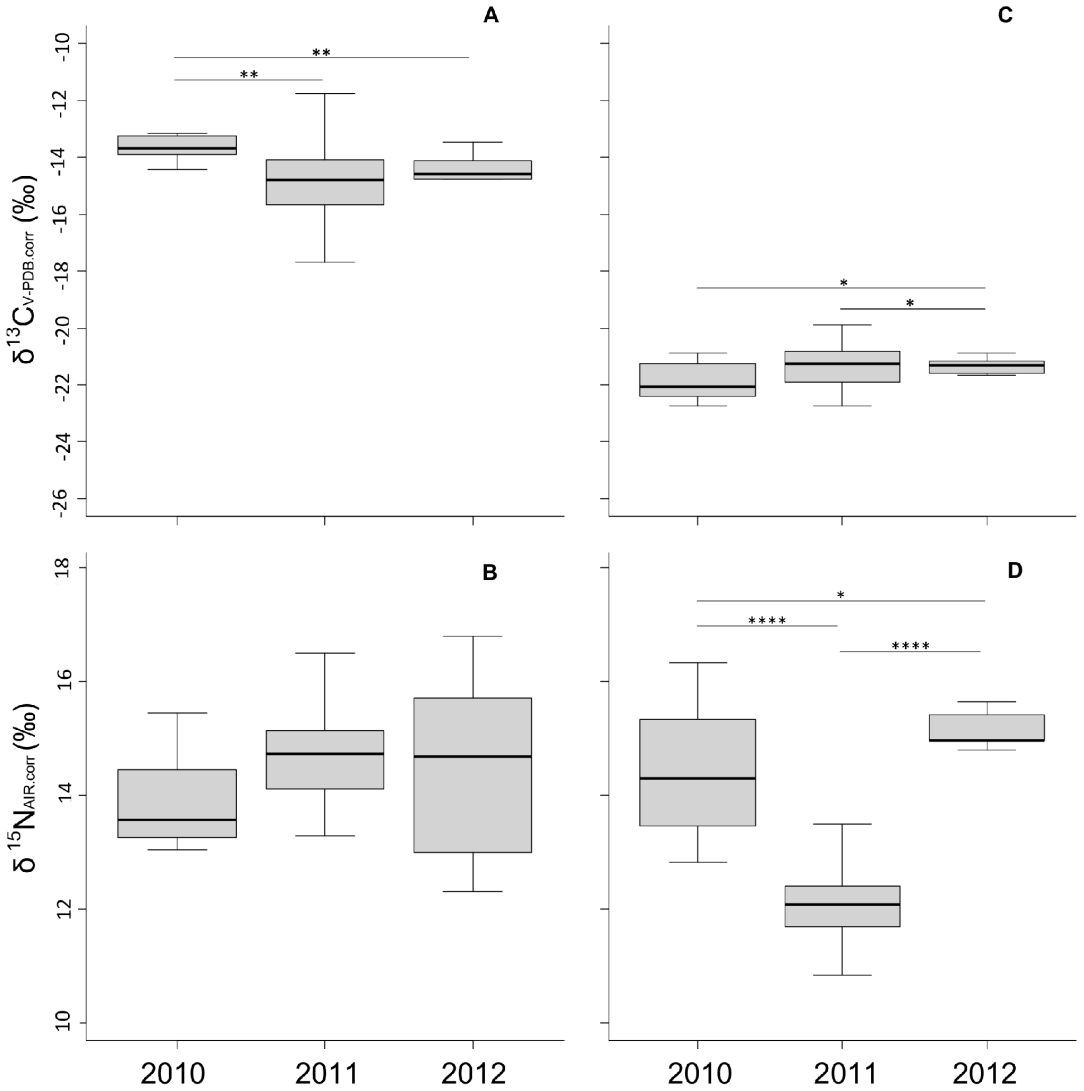
Mean (± s.d) of the yearly mean stable carbon isotope tissue composition of A gemsbok and C springbok. **B and D represent the yearly mean nitrogen isotope composition of gemsbok and springbok tissues, respectively.** The levels of significant differences are shown (*p<0.05, **p<0.01, ***p<0.001, ****p<0.0001).

The mean isotopic mixtures of collected tissues from gemsbok and springbok varied among years (F_2,105_ = 5.42; Pillai = 0.48; p<0.0001; Fig. 2). Gemsbok and springbok differed in their respective isotopic signatures (F_1,105_ = 250.92; Pillai = 0.94; p<0.0001; Fig. 1 & 2) and this difference was consistent over years (F_2,105_ = 5.1; Pillai = 0.46; p<0.0001; Fig. 1 & 2).

Results of our study showed that the mean stable carbon isotope ratio of gemsbok tissues differed between 2010 and 2011 (W = 282; p = 0.0028; Fig. 2 A), between 2010 and 2012 (W = 69; p = 0.0013; Fig. 2A) but not between 2011 and 2012 (W = 157; p = 0.72; Fig. 2A). Data analysis further indicated that the mean nitrogen isotope ratios of gemsbok did not differ between 2010 and 2011 (W = 142; p = 0.45 Fig. 2B), or between 2010 and 2012 (W = 38; p = 0.86; Fig. 2B) or between 2011 and 2012 (W = 176; p = 0.91; Fig. 2B). With respect to the springbok population, our model revealed that the mean stable carbon isotope ratios of the three tissues (blood, liver and muscle) differed between 2010 and 2012 (W = 33; p = 0.015), and between 2011 and 2012 (W = 195; p = 0.043), but not between 2010 and 2011 (W = 163; p = 0.31). Moreover, mean values of nitrogen stable isotope ratios differed between 2010 and 2011 (W = 245; p<0.0001), between 2010 and 2012 (W = 5; p<0.041) as well as between 2011 and 2012 (W = 0, p<0.0001). Simultaneously, we observed a large range of deviating isotopic values within each distinct gemsbok population (Fig. 2A, 2B). Accordingly, we calculated mixing models for both species and for each year separately instead of pooling isotopically similar data from 2011 and 2012.

### Relative Contribution of Potential Food Sources to the Animal Diet

**Figure 3:**
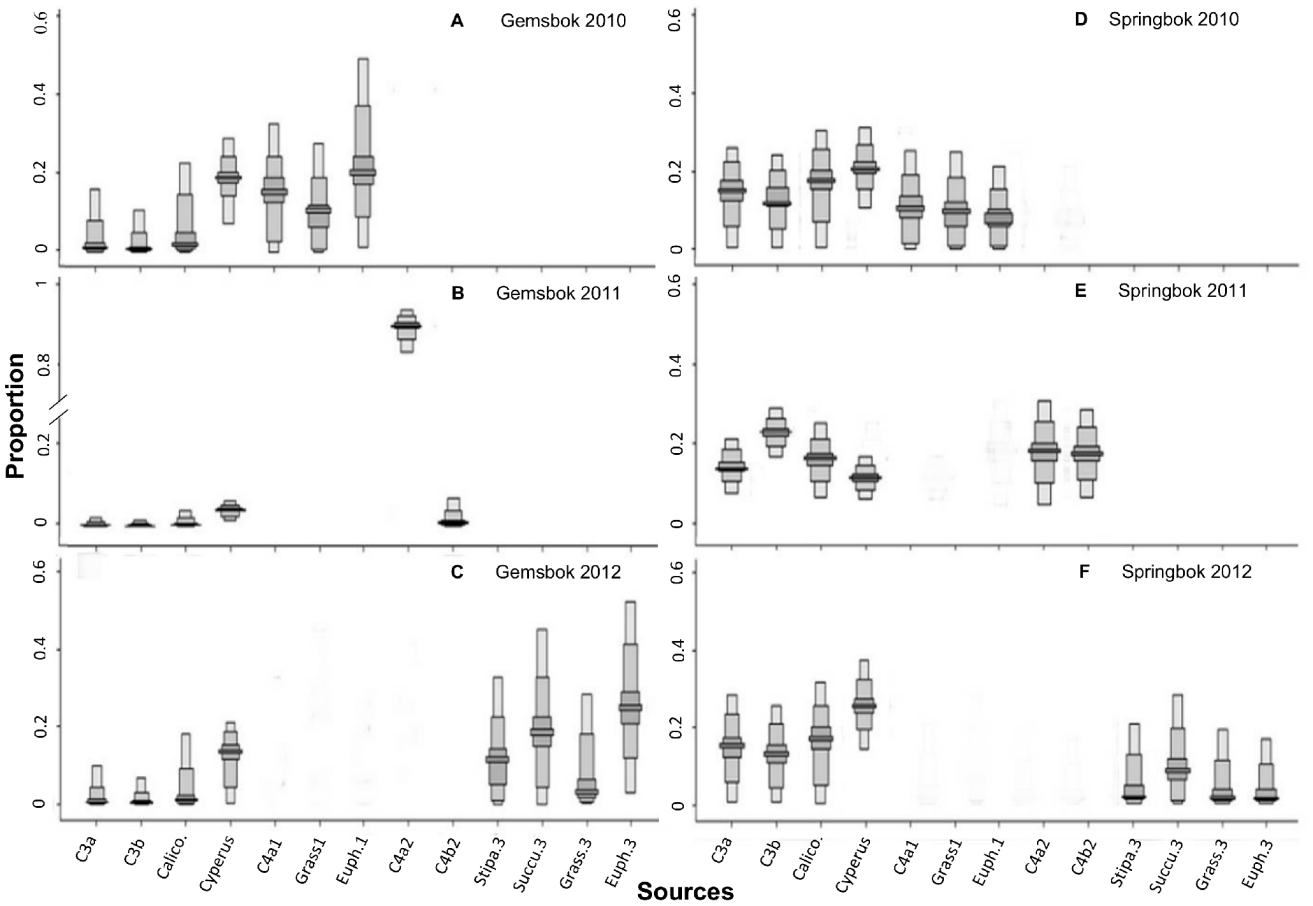
Relative contribution of the potential food sources to the diets of gemsbok (A,B,C) and springbok (D, E, F), as determined by our SIAR isotope mixing model relative to the mean composition of the three metabolically active tissues analysed (blood, liver, muscle) for 2010 (A and D); 2011 (B and E) and 2012 (C and F). The boxplots show the relative proportions of each food source with 95% (dark grey), 75%, 25% and 5% (lightest grey) credibility intervals.

**Figure 4:**
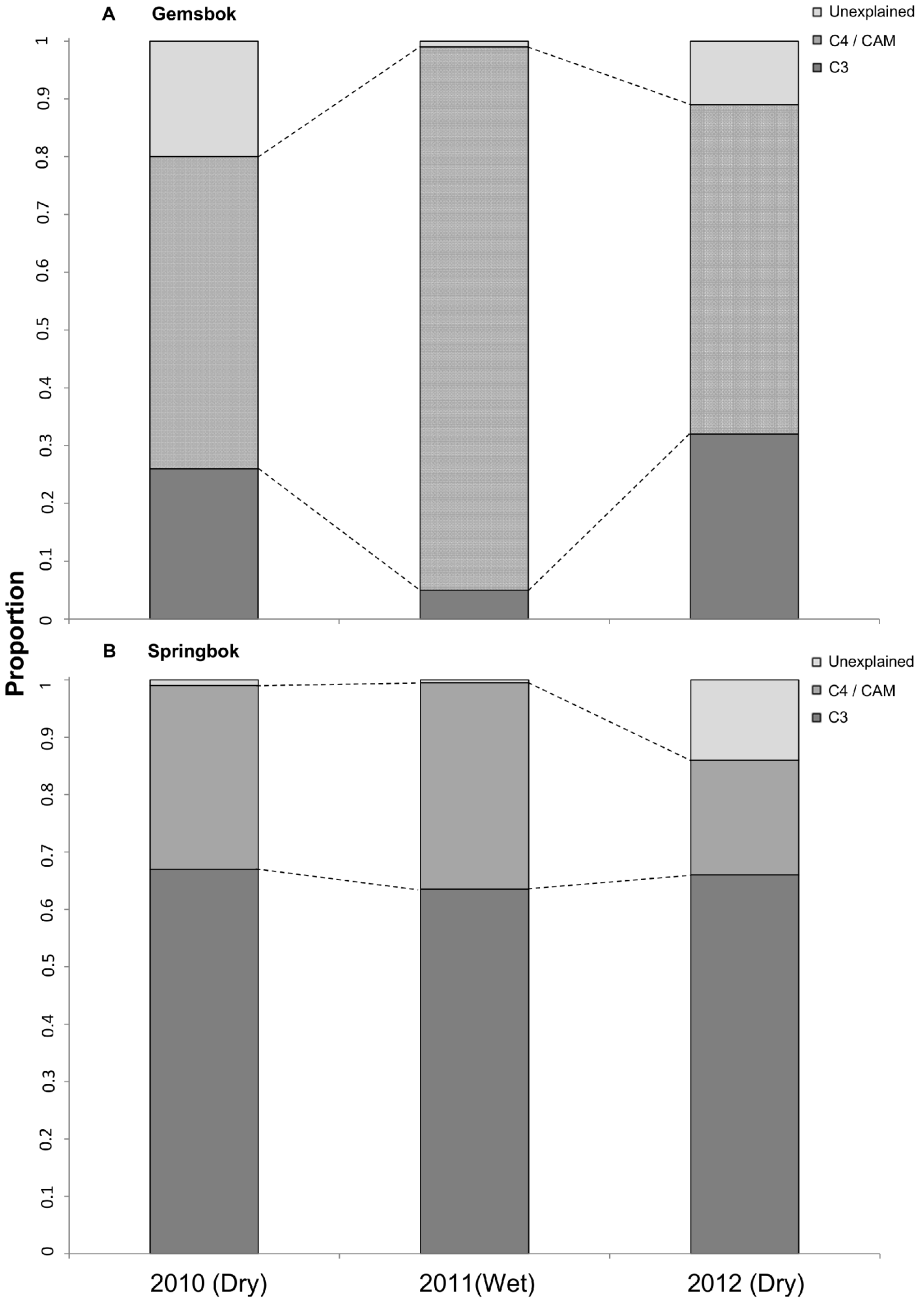
Relative contribution of the potential food sources to the diets of gemsbok (A) and springbok (B), as determined by our SIAR isotope mixing model relative to the mean composition of the three metabolically active tissues analysed (blood, liver, muscle) and for our three focal years. The boxplots show the relative proportions of each food source with C3 plant source (dark grey), C4/CAM plant source and the remaining explained part of the diets (lightest grey).

Using isotopic mixed models (SIAR) for each of the collection years, we estimated the relative contribution of the major food sources to the diets of gemsbok (Fig. 3 A, B & C) and springbok (Fig. 3E; 3F; 3G). For gemsbok, the best model explained 90% of the variation in stable isotope ratios. This model suggested that gemsbok fed on average on 68±21.7% of C4/CAM and on 21±14.2% of C3 plants (Fig. 4 A). Our best model for springbok explained 96% of the variation in stable isotope ratios. According to this analysis, springbok included 65±8% C3 and 29±1.5% C4/CAM plants in their diet (Fig. 4 B) (Mean ± SD of the three year mode for each species). However, our results regarding gemsbok indicate a shift in food resource utilization between dry and wet years. Gemsbok diet included 93% C4/CAM and 5% C3 plants sources in 2011, whereas the diets of 2010 and 2011 were more balanced with respect to the relative contributions of plant types (Fig. 3 A, B & C; Fig. 4 A). Our data also suggest that springbok diets were less variable than those of gemsbok. Indeed, the pattern of resource utilization for springbok did not change from wet to dry years (Fig. 4 B). Our model revealed that the isotopic signature of springbok tissues matched a broad range of plant, with a constant mixed use of C3 and C4/CAM sources and with a preference for C3 plants.

## Discussion

We studied the effect of increased food availability following an intense rain period on the feeding habits of gemsbok and springbok in the arid Kunene region of Namibia. We found that (1) the stable isotopic compositions of C4 and CAM plant sources varied at a local and restricted geographical scale, between years of extreme drought and exceptional rainfall. Further, (2) stable isotope ratios in consumer tissues differed between gemsbok and springbok and respective isotopic compositions of these tissues varied annually according to resource availability. In addition, (3) annual changes reflected the severity of the drought. Lastly, the study indicated that (4) gemsbok were flexible in their diet but specialist feeders when preferential food resources were available. In contrast, springbok were constant generalist feeders. In the next paragraphs, we discuss each finding in detail.

### Plant Isotopic Compositions

The stable isotopic ratios of C4/CAM plants categories varied between years of extreme drought and the intermediate year of unusual rainfall at a local scale. Similar variations in isotopic compositions of plant species have been previously recorded at a much larger spatial scale, encompassing environments with different precipitation patterns [33], [51], [52], [53], [54], [55]. In our study, the variations in C4/CAM plant isotopic signatures were possibly related to the fluctuations in rainfall intensity during the study period. The observed variations in plant isotope compositions were mostly related to variation in δ^15^N of C4/CAM plants, since δ^13^C values of C4 plants are not known to correlate with water availability (e.g. the extent of precipitation) [52], [53]. The large range of ^15^N enrichments in plant matter might be related to varying levels of aridity. Also, δ^15^N might differ between C4/CAM plant species [55], [56], [57], [58]. In our study, we also showed that the perennial *Stipagrostis sp*, (including the endemic *Stipagrostis damarensis*) were enriched in ^15^N compared with less resistant, more ephemeral grass species such as *Eragrostis sp*. This might reflect the fact that *Stipagrostis* is better adapted to grow during relatively dry conditions [54], [55], [56], [58]. Using a combination of *a priori* categories that were based on knowledge and *post hoc* categories that were based on statistical criteria [42], [44], we defined C4/CAM plants categories for each year of the study period. In contrast to C4 and CAM plants, we did not record any inter-annual variations in C3 plant isotope compositions. On the regional scale, C3 plants of arid environment exhibited higher δ^15^N than C3 vegetation of wetter areas [52], [57], [58], [59]. Since we subsumed several species in the various C3 plant categories, it is possible that the resulting higher variation in stable isotope ratios may have obscured inter-annual differences in isotope compositions for the C3 plant categories [52], [53], [55]. Moreover, intra-specific variation in isotopic composition might have also occurred between individuals collected from different micro-habitats, such as river beds, rocky plains or mountain slopes [60], [61]. Yet, we could not control for these effects in our data analysis because the number of putative interfering factors was large in relation to the sample size of our study.

### Animal Tissues Compositions and Inferred Diets

Long-term climate data confirmed that the Torra conservancy of the Kunene region faced a five year drought that ended in early 2011 (Torra Conservancy, Namibian Weather Network [34], Damaraland Camp Weather station). Prior to March 2011, the local ecosystem received only little rain (<80 mm per year) and temperatures reached up to 50°C at sun-exposed places. During such conditions, only a few patchily distributed and dry perennial grasses persist above ground and are thus accessible to ungulates. According to the variation in ^13^C enrichment in animal tissues over the nineteen months study period, gemsbok included more plant resources during the dry than during the wet years. The gemsbok population of the Torra conservancy used leaves from perennial bushes such as *Boscia foetida, Calicorema capitata, Salvadora persica* but seemed to rely more on the resistant evergreen *Cyperus marginatus*. However, the inferred diet of gemsbok included mostly C4/CAM plants with a predominance of *Euphorbia damarana* and a mixture of high ^15^N grasses and succulent plants. Succulent plants are well adjusted to adverse conditions [62] and may represent a useful resource for ungulates during dry periods, because they are rich in water [24]. Yet, our study is the first to demonstrate the significant use of succulent plants by gemsbok (up to 40% of the overall diet), which may explain why gemsboks are relatively independent from drinking water during extended periods of drought. Most interestingly, gemsbok predominantly fed on the evergreen *Euphorbia damarana* during the dry years. This euphorbia is highly toxic and endemic to our study area [63]. Other *Euphorbia* species are used by other herbivores as well, such as browsing Kudu *Tragelaphus strepsiceros* [64], black rhinoceros *Diceros bicornis bicornis* [65] or small antelopes [66] but have never been documented to be utilized by an ungulate that is traditionally considered a grazer. Hence, our result suggests that the gemsbok population of the Kunene region may have evolved physiological abilities that allow them to process or tolerate the highly toxic secondary compounds of *Euphorbia damarana* and consequently to benefit from its high water and nutritious content. In 2011, during our second study period, the local ecosystem received unusually heavy rainfall (>500 mm within two months; Torra conservancy, Damaraland Camp Weather station, [34]). As a consequence, we observed a large increase in flowering perennial and ephemeral grasses with high and low ^15^N values; respectively, which were almost uniformly distributed across various habitats of our study area. During this time, gemsbok consumed these available and relatively easily palatable plants, an observation that is in agreement with earlier studies [67], [68]. However, during the rainy year, gemsbok did not include *Euphorbia damarana* in their diet. Instead, they seemed to feed on a mixture of grasses and succulents.

In 2012, when rainfall decreased in intensity by more than half, we observed an increase in ^13^C enrichment in the gemsbok tissues. From this, our stable isotope mixing model inferred an increased contribution of *Euphorbia damarana* and succulent plants to the gemsbok diet. Our stable isotope mixing model suggested an intermediate use of C4/CAM and C3 plants as food, meaning that although animals are using both resource types, their diets are biased toward C4 and CAM plants. The evergreen *Cyperus marginatus* as well as *Calicorema capitata* and other perennial shrubs such as *Boscia foetida* and *Salvadora persica* were used as food; probably in response to the shortage in *Stipagrostis sp.* and low ^15^N, less resistant grasses. Similar to 2010, *Euphorbia damarana* represented one of the most utilized food items for gemsbok.

The diet of springbok was more constant over the nineteen months of our study period, with fewer variations in C3 versus C4/CAM resource contributions between years. However, we observed an enrichment of ^13^C and a depletion of ^15^N in the mean isotope tissue compositions between 2010 and 2011. This can be explained by a dietary switch from *Cyperus marginatus/Calicorema capitata* mixture in 2010 to plants of the category C3b (shrubs & trees) in 2011. The depletion of springbok tissue in ^15^N during 2011 could be explained by the much larger proportion of plants from the categories “C4a2” (*Stipagrostis sp*. & succulent plants) and “C4b2” (low ^15^N grasses & *Euphorbia damarana*) in the diet of springbok. Both C4 and CAM categories had lower δ^15^N values in contrast to *Calicorema capitata* and *Cyperus marginatus* food sources. In 2012, we observed that springbok tissues were enriched in ^13^C and depleted in ^15^N compared to 2010 and 2011. This indicated the combined and predominant use of *Cyperus marginatus, Calicorema capitata*, C3a and C3b food source categories as grasses availability decreased.

In this study, we demonstrated a high dietary plasticity of gemsbok during times of fluctuating primary productivity and water availability. Indeed, the gemsbok populations in our study area ingested a broad range of plants, including mostly C3 and C4/CAM plants during years of extremely low rainfall (2010 and 2012) but specialized on grasses during the year of exceptional heavy rainfall (2011) with high primary productivity. Hence, the reduced availability of grass plant matter during prolonged dry periods clearly led gemsbok to supplement their diet with alternative food sources. Contrary to our predictions, gemsbok was able to successfully expand their dietary niche when needed and thus did not move away from our study area. We also observed a large range of deviating isotopic values in stable isotope ratios for gemsbok, possibly suggesting some individual feeding preferences or movements on a larger scale. As aridity increased and food resource availability decreased, animals might have travelled excessively and avoided aggregations [69]. Consequently, individuals from the same population might have visited different habitats with contrasting isotopic baselines [69], [70]. Individuals of the same population may have therefore specialized on a specific food mixture during the drought period in order to reduce intra-specific competition. Since sample sizes were low for animal tissues in 2010 and 2012, we were not able to elucidate better the underlying causes for isotopic variation within a given year. Hence, further studies, including a larger number of samples per individual over an extended lapse of the animal lifetime are needed to explicitly document this potential phenomenon of individual isotopic specialization. Springbok were mixed, intermediate feeders of C3 plants. However, the important shift of their mean tissues isotopic composition from one year to another, in conjunction with the small standard deviation of the overall C3, C4 and CAM resources used revealed a lower dietary plasticity in response to changes in precipitation patterns. Additionally, the smaller range of deviating isotopic values implies that individuals of the local population were mostly feeding on the same mixture of plants. In contrast to our expectation, springbok seem to have a lower dietary plasticity than gemsbok possibly for the simple reason that they might not need it.

In this study we observed distinct dietary strategies in two ungulate species with different body size. Gemsbok and springbok preferred different food sources at any time of our study period and do not necessarily overlap in resource use. This mechanism of resource partitioning may facilitate the coexistences of these two ungulate species [71]. Moreover gemsbok might facilitate the access to high quality grasses during increased primary productivity by cropping off dried plants, allowing springbok to easily access low height young green sprouts, which in turn stimulate plants to grow faster and higher [72], [73], [74]. Possibly gemsbok are then rewarded with a freshly grown plant source with adequate palatable height. To conclude, we inferred from stable isotope ratios of plants and animal tissues the contrasted diets of gemsbok and springbok in the arid Kunene environment. We successfully demonstrated a radical shift in gemsbok diet between years of different precipitation rhythms, while springbok diet remained constant, but intrinsically varied.
