# Correction: Dietary Plasticity of Generalist and Specialist Ungulates in the Namibian Desert: A Stable Isotopes Approach

**DOI:** 10.1371/annotation/89fe804f-20cc-40cc-99af-d42751676d36

**Published:** 2013-09-11

**Authors:** David Lehmann, John Kazgeba Elijah Mfune, Erick Gewers, Johann Cloete, Conrad Brain, Christian Claus Voigt

There was information omitted from the Author Contributions. The correct version of this section is available below.

Conceived and designed the experiments: DL CCV. Performed the experiments: DL EG JC. Analyzed the data: DL. Contributed reagents/materials/analysis tools: DL JKEM JC EG CB CCV. Wrote the paper: DL JKEM CCV. Logistics: DL JKEM EG CB. Permits: DL. Data collection: DL EG JC. 

